# Long term results of a prospective multicenter observational study on the use of anti-human T-lymphocyte immunoglobulin (ATLG) in unrelated donor transplantation (ATOS study)

**DOI:** 10.1038/s41409-024-02264-9

**Published:** 2024-03-16

**Authors:** Jürgen Finke, Claudia Schmoor, Francis Ayuk, Justin Hasenkamp, Mareike Verbeek, Eva-Maria Wagner, Harald Biersack, Kerstin Schäfer-Eckart, Dominik Wolf, Gernot Stuhler, Roland Reibke, Christoph Schmid, Martin Kaufmann, Matthias Eder, Hartmut Bertz, Olga Grishina

**Affiliations:** 1 University Medical Center and Medical Faculty, University of Freiburg, Breisgau, Germany; 2 Clinical Trials Unit, Medical Center and Faculty of Medicine, University of Freiburg, Breisgau, Germany; 3 University Medical Center Hamburg-Eppendorf, Hamburg, Germany; 4 University Medical Center Göttingen, Göttingen, Germany; 5 Technical University of Munich, School of Medicine, Klinikum rechts der Isar, Clinic and Policlinic for Internal Medicine III. München, Munich, Germany; 6 University Medical Center Mainz, Mainz, Germany; 7 Universitätsklinikum Schleswig-Holstein Campus Lübeck, Lübeck, Germany; 8 Medical Center Nürnberg Nord, Nürnberg, Germany; 9 Medical Clinic 3, Universitätsklinikum Bonn, Bonn, Germany; 10 DKD Helios Klinik Wiesbaden, Wiesbaden, Germany; 11 University Medical Center LMU München, Munich, Germany; 12 Klinikum Augsburg, Augsburg, Germany; 13 Robert Bosch Krankenhaus Stuttgart, Stuttgart, Germany; 14 University Medical Center Hannover, Hannover, Germany; 15 Present Address: Klinikum Kulmbach, Bavaria, Germany; 16 Present Address: Dpt. Hematology and Oncology, Comprehensive Cancer Center Innsbruck (CCCI), Innsbruck, Austria; 17 Present Address: Universätsklinikum Würzburg, Würzburg, Germany

**Keywords:** Phase IV trials, Leukaemia, Acute myeloid leukaemia

## Abstract

ATOS is a prospective observational study evaluating the outcome of patients receiving anti-human T-lymphocyte immunoglobulin (ATLG) in unrelated donor transplantation. Primary endpoint was severe GvHD and relapse-free survival (SGRFS). GvHD prophylaxis consisted of ATLG and CSA/ MTX or MMF. Outcome was compared to the ATLG arm of our prospective randomized phase III multicenter trial trial (RCT) [1, 2]. 165 patients, median age 54 (18; 77) years, with haematological malignancies with early (45.5%), intermediate (17.6%), and advanced (37.0%) disease were included. ATLG dose differed between centers according to local practise (median total ATLG dose of 46 (IQR 32–60, range 15–91) mg/kg). Median follow-up was 70 months. Estimated probabilities at 5 years follow up were for SGRFS 0.27, OS 0.52, DFS 0.43, NRM 0.23, relapse 0.34, acute GvhD °III/IV 0.13, severe chronic GvHD 0.27. OS rates differed dependent on disease status. An effect of the given ATLG dose could not be separated from potential center effects. Despite higher age and more advanced disease in ATOS, outcome was similar to the ATLG arm of our RCT. This long-term, multicenter, experience in routine clinical practice confirms the GvHD-protective effect of ATLG without compromising relapse and non-relapse mortality rates.

Clinical Trial Registry: German clinical trials register DRKS00004581.

## Introduction

Chronic graft-versus-host disease (GvHD) is a major cause of non-relapse mortality (NRM) and of morbidity following allogeneic hematopoietic stem cell transplantation (SCT). Incidence and prevalence of cGvHD are related to risk factors like patient’s age, donor age, use of peripheral blood stem cells instead of bone marrow, matching degree, etc. Anti-human T-lymphocyte immunoglobulin (ATLG) might lower the incidence of GvHD [3, 4]. We conducted a prospective, randomised, multicentre, open-label, phase III trial to compare standard GvHD prophylaxis with cyclosporine A and methotrexate with or without additional pretransplant ATLG (Grafalon, previously ATG-FRESENIUS S) (given 20 mg/kg/day, days −3 to −1) in unrelated donor hematopoietic cell transplantation after myeloablative conditioning (MAC) [1]. Long-term follow-up in our randomized phase III multicenter trial (RCT) demonstrated that the addition of ATLG resulted in decreased incidence of acute and chronic GvHD without an increase in relapse or non-relapse mortality, and without compromising overall survival [2, 5, 6].

ATOS is a subsequent prospective non-interventional observational study (NIS) with the aim to evaluate the outcome of patients receiving ATLG in unrelated donor transplantation in routine clinical practice without the selective measures of a clinical trial.

## Methods

### Study design and participants

ATOS is a prospective, national, multicenter, non-interventional, single-arm, observational study. Patients’ characteristics and the outcome of patients were compared to the outcome of the patients in the ATLG treatment arm in our RCT [1, 2]. A further aim was to evaluate the relationship of patient, disease and treatment characteristics (prognostic factors) with outcome. Adult patients who suffer from hematological malignancies undergoing SCT from an unrelated donor who were going to receive ATLG (ATG-Fresenius S until December 2015/Grafalon from January 2016) as GvHD prophylaxis were eligible (further details in Supplementary appendix).

### Procedures

Before start of treatment, patients were prospectively registered centrally at the clinical trials unit of the Medical Center, University of Freiburg, Germany. Treatment and procedures were performed according to center standards.

Patients gave written consent, and the study was approved by the German competent authority and by independent ethics committee, and was done in accordance with good clinical practice guidelines, the Declaration of Helsinki, and national regulatory requirements. The study was registered in the German clinical trials register (DRKS00004581). The sponsor was Neovii.

After completion of the study, it was considered relevant to obtain long-term follow-up data later than 12 months after SCT regarding GvHD, relapse and survival. Therefore, in July 2021, the most up-to-date follow-up data on clinical endpoints for 118 patients being alive at study end 12 months after SCT were requested from the DRST (German Registry for Stem Cell Transplantation). These were data on chronic GvHD, relapse, and overall survival. Additionally, detailed data on the conditioning regimen applied before SCT were requested from the DRST, as this had not been collected within the study. Conditioning regimens were grouped into four categories (A: similar to Cy-TBI, B: similar to Bu-Cy, C: similar to Bu-Flu, D: other regimens) according to conditioning regimens used in the ATLG-US-study [7].

### Statistical analysis

Statistical analyses were performed in the full analysis set including all patients who received at least one dose of study drug ATLG and for whom relapse status in the course of follow up was available. The primary endpoint was severe GvHD and relapse-free survival (SGRFS), defined as survival free of severe GvHD (acute GvHD III-IV or severe chronic GvHD) and free of relapse. Chronic GvHD was classified as severe, if it was rated as extensive according to the Seattle criteria or as severe according to the NIH criteria. Secondary endpoints were engraftment of ANC and platelets, acute GvHD, chronic GvHD, relapse, non-relapse mortality (NRM), disease-free survival (DFS), overall survival (OS), new malignancies, quality-of-life, employment status, hospital stays, and safety (adverse drug reactions, serious adverse drug reactions, severe infections). For the analysis of the combined endpoint SGRFS, DFS and OS, the probabilities were estimated by the Kaplan-Meier method. For the analysis of time-to-event variables, for which competing events have to be considered, the probabilities were estimated by the Aalen-Johansen estimator. Comparisons between patient groups were performed with Cox regression models used also for the estimation of adjusted event probabilities. The effects of prognostic factors on outcome were analyzed in univariate and multiple Cox regression models. Additionally, the outcome of the patients of the ATOS study was compared to the outcome of the patients in the ATLG treatment arm in our RCT. For details on sample size planning and further details of the statistical methods see Supplementary appendix.

## Results

### Patient and treatment characteristics

Between May 2013 and March 2015, thirteen transplant centers registered 172 patients for the study. Seven patients were excluded from the full analysis set, as five patients did not receive ATLG and for two patients no information on relapse was available. Twelve patients older than 65 years were included in the study, these were not excluded from the analyses. Thus, all analyses included 165 patients (Table 1).

**Table 1:** Baseline patient and disease characteristics in ATOS as compared to the ATLG treatment arm of our RCT.

Baseline characteristics	ATOS *N* = 165	ATLG (RCT) *N* = 103
Patient age (median, [min; max])	54 [18; 77]	40 [18; 60]
< 40 years	26 (15.8%)	47 (45.6%)
≥ 40 years	139 (84.2%)	56 (54.4%)
Donor age (median, [min; max])	32 [19; 67]	35 [20; 58]
< 40 years	112 (67.9%)	62 (66.0%)
≥ 40 years	53 (32.1%)	32 (34.0%)
Patient/Donor sex		
Patient male/Donor female	20 (12.2%)	14 (13.9%)
Other	144 (87.8%)	87 (86.1%)
Patient CMV status		
Positive	83 (50.3%)	66 (64.1%)
Negative	82 (49.7%)	37 (35.9%)
Donor CMV status		
Positive	66 (40.0%)	48 (46.6%)
Negative	99 (60.0%)	55 (53.4%)
Type of primary disease		
AML	89 (53.9%)	55 (53.4%)
ALL	24 (14.5%)	37 (35.9%)
MDS	17 (10.3%)	5 (4.9%)
CML	3 (1.8%)	6 (5.8%)
OMF	6 (3.6%)	0 (0.0%)
Other	26 (15.8%)	0 (0.0%)
Disease status		
Early	75 (45.5%)	64 (62.1%)
Intermediate	29 (17.6%)	15 (14.6%)
Advanced	61 (37.0%)	24 (23.3%)
Conditioning intensity		
Myeloablative (MAC)	81 (50.9%)	103 (100.0%)
Reduced intensity conditioning	78 (49.1%)	0 (0.0%)
(RIC)		
Other/missing	6	
Time from primary diagnosis of current disease to transplantation		
< 1 year	121 (73.3%)	84 (81.6%)
≥ 1 year	44 26.7%)	19 (18.4%)
Stem cell source		
Bone marrow	6 (3.6%)	21 (20.4%)
Peripheral blood	159 (96.4%)	82 (79.6%)
HLA mismatch (10/10, 4-digit)	37 (22.4%)	31 (33.7%)
ATLG dose (median, [min; max])		
absolute given total dose per kg	46 [15; 91]	60 [19; 65]
< 45 mg/kg	81 (49.1%)	6 (5.8%)
≥ 45 mg/kg	84 (50.9%)	97 (94.2%)

GvHD prophylaxis consisted of calcineurin inhibitors, mainly CSA (*N* = 154, 93%) with MTX or MMF and ATLG. Different dosing regimens were administered according to current local practice of centers. Median total ATLG dose was 46 mg/kg (IQR 32–60 mg/kg, range 15–91 mg/kg). A high variation of given total ATLG dose was to be observed between centers. Lowest total ATLG dose was given in the largest center with 40 patients (called center 1 with a median dose of 32 mg/kg, range 15–36 mg/kg), whereas median given total dose was around 60 mg/kg in most of the other centers (for details see Supplementary appendix Table S1).

With regard to disease status, 75 (45.5%) patients had early, 29 (17.6%) had intermediate, and 61 (37%) had advanced disease. Early disease was more frequent in AML and ALL, in patients with MAC, in patients with a mismatched transplant, and in patients with less than one year from primary diagnosis of current disease to transplantation. With regard to HLA-match, a transplant from a mismatched donor was given to 37 patients, 128 received a transplant from a matched donor. A mismatched transplant was given more often to younger patients, in cases of male patients with female donor, in patients with AML and MDS, and in patients with early disease status. With regard to conditioning intensity, 81 patients received MAC, 78 patients received RIC, five patients received other non-myeolablative regimens, and for one patient it was unknown. MAC was more applied in younger patients, in patients with ALL, and in patients with early or intermediate disease status. With regard to conditioning regimen group, 40 patients received regimen group A similar to Cy-TBI (29 MAC, 9 RIC, 2 other), 38 patients received regimen group B similar to Bu-Cy (28 MAC, 10 RIC), 64 patients received regimen group C similar to Bu-Flu (13 MAC, 50 RIC, 1 other), and 22 patients received regimen group D other regimens (11 MAC, 9 RIC, 2 other). Conditioning regimen group A was applied more often in younger patients, mainly in patients with ALL, more often in patients with early disease status, and in patients who received a transplant from a mismatched donor.

As compared to the 103 patients in the ATLG arm of our RCT [1], patients in ATOS were older, their disease status was more advanced, reduced intensity conditioning (RIC) was given, rates of transplantation with HLA mismatch (10/10, 4-digit) and with bone marrow were lower, and given median ATLG dose was lower (see Table 1).

### Clinical efficacy endpoints

The cumulative incidence of engraftment (ANC > 1.0/nL) at month 3 after SCT was 0.93, 95% CI [0.90; 0.97], and that of engraftment (platelets > 50/nL) at month 12 after SCT was 0.88, 95% CI [0.82; 0.93].

Median follow-up of patients with regard to GvHD, relapse, and survival was 5.8 years (IQR 3.9–6.3 years). Estimated probabilities at 5 years follow up were for SGRFS 0.27, OS 0.52, DFS 0.43, NRM 0.23, relapse 0.34, acute GvhD °III/IV 0.13, severe chronic GvHD 0.27 (see Table 2). For a detailed comparison to the ATLG arm of our RCT [1] see supplementary appendix.

**Table 2:** Estimated probabilities of different endpoints over time in the ATOS study as compared to the ATLG arm of our RCT.

Endpoint	Year	Incidence / rate with 95%-CI	
		ATOS *N* = 165	ATLG (RCT) *N* = 103
Severe GvHD and relapse-free survival (SGRFS)	1	0.41 (0.34–0.49)	0.49 (0.39–0.58)
	5	0.27 (0.20–0.34)	0.34 (0.25–0.43)
Acute GvHD any	1	0.56 (0.49–0.64)	0.56 (0.48–0.67)
Acute GvHD III-IV	1	0.13 (0.09–0.19)	0.12 (0.07–0.20)
Chronic GvHD any	1	0.30 (0.23–0.38)	0.28 (0.20–0.39)
	5	0.42 (0.34–0.51)	0.31 (0.23–0.42)
Chronic GvHD severe	1	0.22 (0.16–0.29)	0.11 (0.06–0.20)
	5	0.27 (0.20–0.35)	0.14 (0.08–0.23)
Relapse	1	0.22 (0.16–0.29)	0.22 (0.16–0.32)
	5	0.34 (0.27–0.42)	0.35 (0.27–0.46)
Non-relapse mortality	1	0.17 (0.12–0.24)	0.18 (0.12–0.27)
	5	0.23 (0.18–0.31)	0.21 (0.14–0.30)
Disease-free survival	1	0.61 (0.54–0.69)	0.60 (0.51–0.70)
	5	0.43 (0.35–0.51)	0.44 (0.35–0.54)
Overall survival	1	0.73 (0.66–0.80)	0.69 (0.60–0.78)
	5	0.52 (0.44–0.60)	0.50 (0.40–0.60)

**Fig. 1:**
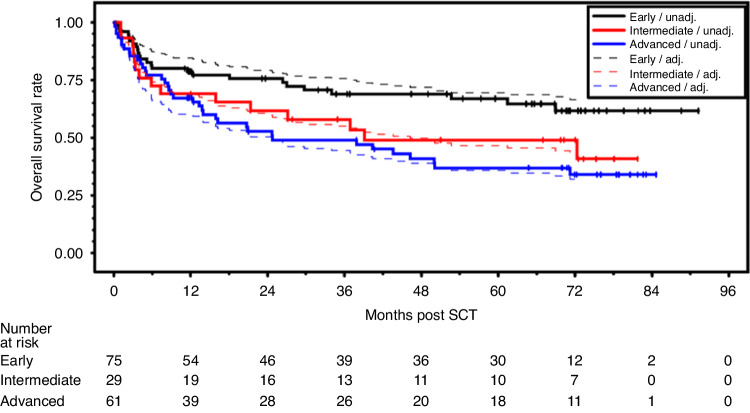
Probability of OS by disease status. Estimated probability of overall survival (OS) by disease status - unadjusted and from Cox regression model adjusted for center, type of disease, conditioning intensity, time from primary diagnosis of current disease to transplantation, HLA-mismatch.

**Fig. 2:**
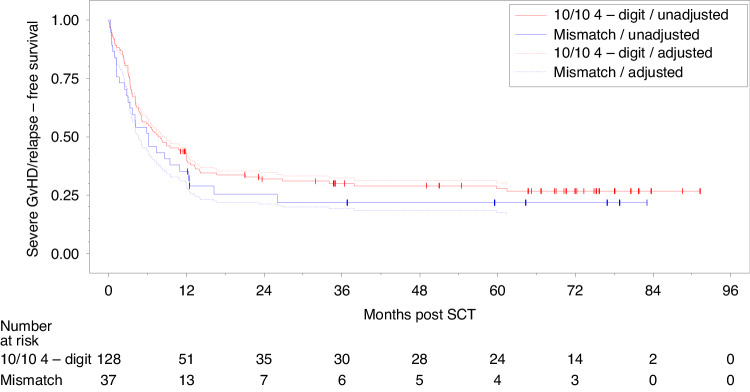
Probability of SGRFS by HLA-mismatch. Estimated probability of severe GvHD and relapse-free survival (SGRFS) by HLA-mismatch - unadjusted and from Cox regression model adjusted for center, patient age, patient and donor sex, type of disease, disease status, conditioning regimen.

OS rates differed dependent on disease status with 0.67, 0.49 and 0.37 for early, intermediate and advanced disease at 5 years (see Fig. 1). HRs adjusted for center and the above mentioned factors were intermediate vs. early 2.28, 95%-CI (1.11–4.68), and advanced vs. early 3.24, 95%-CI (1.70–6.16), *p* = 0.0016. With regard to HLA-match, GvHD incidences were slightly higher with a mismatch leading to a slightly decreased SGRFS rate with an HR adjusted for center (centers with less than 20 patients combined) and the above mentioned factors was 1.54, 95%-CI (0.96–2.47), *p* = 0.073 (see Fig. 2). With regard to conditioning intensity and conditioning regimen, no relevant relationships to clinical endpoints were observed.

**Fig. 3:**
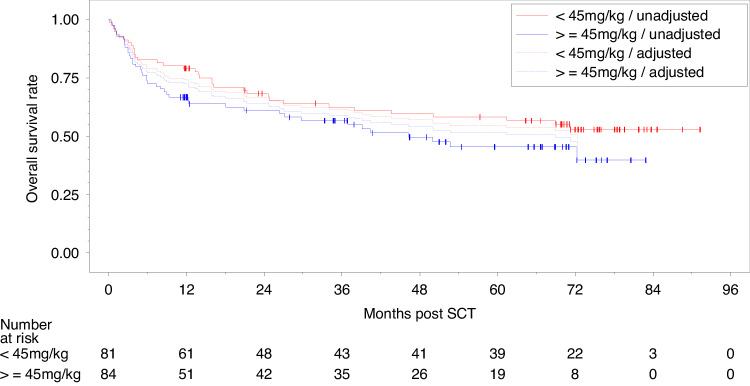
Probability of OS by ATLG-dose. Probability of overall survival (OS) by ATLG-dose - unadjusted and from Cox regression model adjusted for center, patient age, donor age, CMV status of patient, type of disease, disease status, time from primary diagnosis of current disease to transplantation, HLA-mismatch.

Next, we analyzed the impact of the given ATLG dose on disease-relevant outcome parameters after transplantation. Figure 3 shows a slightly higher OS rate for patients treated with less than 45 mg/kg, which was chosen as cut-off dose, but after adjustment for center and prognostic factors, there was almost no difference. Noteworthy, the ATLG dose differed largely between centers, as shown in Table S1 (supplementary appendix). All 40 patients in the largest center 1 received less than 45 mg/kg, whereas 84 (67%) of 125 patients from the other centers received at least 45 mg/kg ATLG. As a consequence, the effect of the given ATLG dose cannot be separated from potential center effects. As shown in Fig. S1 (supplementary appendix), the highest OS rate were observed for patients from center 1 treated with about 30 mg/kg whereas OS rates of patients from other centers treated with less than 45 mg/kg and with at least 45 mg/kg were almost identical. This was true even after adjustment for other relevant prognostic factors. Consequently, no definite conclusions on the effects of the ATLG-dose can be drawn from this real-world data-set.

For a detailed outcome comparison to the ATLG arm of our RCT [2] see Table 2 and supplementary appendix Table S2, Figs. S2 and S3. In summary, the results are similar with the exception of a higher rate of severe chronic GvHD in this study as compared to the RCT, which may be due to different reporting.

### New malignancies, hospital stays, quality of life, adverse drug reactions

Out of 165 study patients, two cases (one fatal) of lymphoproliferative disorders (PTLD) on days 55 and 74 after SCT were reported, in comparison to 5 (3 fatal) of 103 patients in the ATLG arm of our RCT [1]. Weekly PCR-testing for EBV copies was mandatory in ATOS, in contrast to the previous RCT, recruiting patients between 2003 and 2007. In ATOS, another reported malignancy was one recurrence of breast cancer on day 111 after SCT.

For further results on hospital stays, quality of life, adverse drug reactions, severe infections, and reasons of death see Supplementary appendix.

## Discussion

Here we present the long-term outcome of a prospective non-interventional observational study with ATLG in unrelated donor stem cell transplantation. The experience in day-to-day clinical practice confirms the results shown in our randomized trial, namely the GvHD protective effect of ATLG without compromising NRM or relapse rates. This outcome is of special interest because the reassuring results are obtained in a subsequent time period with differences in parameters relevant for outcome: patients were older, had a more advanced disease status, more patients received reduced-intensity conditioning, HLA 10/10 match and PBSC transplantation were more frequent, and given ATLG dose was lower with a median of 46 mg/kg (range 15–91 mg/kg).

Since dosing of ATLG differed between centers a conclusion on the effect of the ATLG dose cannot be drawn because it is confounded by center effects (see supplementary appendix, Table S1, Fig. S1). Even with 165 patients included in this study, there are clear limitations, as many endpoints and many factors result in multiple testing (no control of false positive results). Additionally, no causal interpretation of relationship between factors and endpoints is possible because of confounding factors.

Comparing trials testing the role of anti T cell globulins for GvHD prevention, it should be noted, that in the present ATOS study as well as in our pivotal randomized trial [1] leukemia patients with active disease were included, whereas in all other randomized trials leukemia patients were only included in complete remission (CR1 or CR2) [7–10].

For GvHD prevention alternatives to ATG are available and extensively in use, namely abatacept [11], and post transplant cyclophosphamide (PT-CY) [12]. GvHD prophylaxis with PT-CY originally had been established for matched as well as haplo-identical transplantation with bone marrow for patients in complete remission [12]. Application in patients with active disease resulted in excessive relapse rates, which led to the switch of graft source to peripheral blood-derived hematopoietic cells in many centers [13]. With the observation of more GvHD when using PT-CY after PBSC transplantation, several centers combined PT-CY with additional ATG.

What is best for GvHD prevention? There may be several solutions depending on disease and patient-related factors. For the direct comparison, the results of the ongoing Grappa multicenter randomized trial (EudraCT: 2021-000853-17, PI.: J. Schetelig) comparing PT-CY with ATLG 30 mg/kg, both in combination with Tacrolimus and Mycophenolate Mofetil in matched and mismatched unrelated donor transplantation for patients with AML or MDS, may give an answer.

For now, ATLG is a valid tool for GvHD prevention and long-term severe-GvHD- and relapse-free survival in unrelated donor transplantation.

### Supplementary information

ATOS supplement data

## Data Availability

The datasets generated during and/or analysed during the current study are not publicly available.

## References

[1] Finke J, Bethge WA, Schmoor C, Ottinger HD, Stelljes M, Zander AR (2009). Standard graft-versus-host disease prophylaxis with or without anti-T-cell globulin in haematopoietic cell transplantation from matched unrelated donors: a randomised, open-label, multicentre phase 3 trial. Lancet Oncol.

[2] Finke J, Schmoor C, Bethge WA, Ottinger H, Stelljes M, Volin L (2017). Long-term outcomes after standard graft-versus-host disease prophylaxis with or without anti-human-T-lymphocyte immunoglobulin in haemopoietic cell transplantation from matched unrelated donors: final results of a randomised controlled trial. Lancet Haematol.

[3] Baron F, Mohty M, Blaise D, Socie G, Labopin M, Esteve J (2017). Anti-thymocyte globulin as graft-versus-host disease prevention in the setting of allogeneic peripheral blood stem cell transplantation: a review from the Acute Leukemia Working Party of the European Society for Blood and Marrow Transplantation. Haematologica.

[4] Kumar A, Reljic T, Hamadani M, Mohty M, Kharfan-Dabaja MA (2019). Antithymocyte globulin for graft-versus-host disease prophylaxis: an updated systematic review and meta-analysis. Bone Marrow Transpl.

[5] Finke J, Schmoor C, Bethge WA, Ottinger HD, Stelljes M, Zander AR (2012). Prognostic factors affecting outcome after allogeneic transplantation for hematological malignancies from unrelated donors: results from a randomized trial. Biol Blood Marrow Transpl.

[6] Socie G, Schmoor C, Bethge WA, Ottinger HD, Stelljes M, Zander AR (2011). Chronic graft-versus-host disease: long-term results from a randomized trial on graft-versus-host disease prophylaxis with or without anti-T-cell globulin ATG-Fresenius. Blood.

[7] Soiffer RJ, Kim HT, McGuirk J, Horwitz ME, Johnston L, Patnaik MM (2017). Prospective, Randomized, Double-Blind, Phase III Clinical Trial of Anti-T-Lymphocyte Globulin to Assess Impact on Chronic Graft-Versus-Host Disease-Free Survival in Patients Undergoing HLA-Matched Unrelated Myeloablative Hematopoietic Cell Transplantation. J Clin Oncol.

[8] Bacigalupo A, Lamparelli T, Bruzzi P, Guidi S, Alessandrino PE, di Bartolomeo P (2001). Antithymocyte globulin for graft-versus-host disease prophylaxis in transplants from unrelated donors: 2 randomized studies from Gruppo Italiano Trapianti Midollo Osseo (GITMO). Blood.

[9] Walker I, Panzarella T, Couban S, Couture F, Devins G, Elemary M (2016). Pretreatment with anti-thymocyte globulin versus no anti-thymocyte globulin in patients with haematological malignancies undergoing haemopoietic cell transplantation from unrelated donors: a randomised, controlled, open-label, phase 3, multicentre trial. Lancet Oncol.

[10] Kroger N, Solano C, Wolschke C, Bandini G, Patriarca F, Pini M (2016). Antilymphocyte Globulin for Prevention of Chronic Graft-versus-Host Disease. N. Engl J Med.

[11] Watkins B, Qayed M, McCracken C, Bratrude B, Betz K, Suessmuth Y (2021). Phase II Trial of Costimulation Blockade With Abatacept for Prevention of Acute GVHD. J Clin Oncol.

[12] Rimando JC, McCurdy SR, Luznik L. How We Prevent GVHD in High Risk Patients: Post Transplant Cyclophosphamide and Beyond. Blood 2022. 10.1182/blood.2021015129 10.1182/blood.2021015129 35405017

[13] Battipaglia G, Boumendil A, Labopin M, Ciceri F, Tischer J, Stelljes M (2019). Unmanipulated haploidentical versus HLA-matched sibling allogeneic hematopoietic stem cell transplantation in relapsed/refractory acute myeloid leukemia: a retrospective study on behalf of the ALWP of the EBMT. Bone Marrow Transpl.

