# Correction: Long term results of a prospective multicenter observational study on the use of anti-human T-lymphocyte immunoglobulin (ATLG) in unrelated donor transplantation (ATOS study)

**DOI:** 10.1038/s41409-024-02274-7

**Published:** 2024-04-23

**Authors:** Jürgen Finke, Claudia Schmoor, Francis Ayuk, Justin Hasenkamp, Mareike Verbeek, Eva-Maria Wagner, Harald Biersack, Kerstin Schäfer-Eckart, Dominik Wolf, Gernot Stuhler, Roland Reibke, Christoph Schmid, Martin Kaufmann, Matthias Eder, Hartmut Bertz, Olga Grishina

**Affiliations:** 1 University Medical Center and Medical Faculty, University of Freiburg, Breisgau, Germany; 2 Clinical Trials Unit, Medical Center and Faculty of Medicine, University of Freiburg, Breisgau, Germany; 3 University Medical Center Hamburg-Eppendorf, Hamburg, Germany; 4 University Medical Center Göttingen, Göttingen, Germany; 5 Technical University of Munich, School of Medicine, Klinikum rechts der Isar, Clinic and Policlinic for Internal Medicine III. München, Munich, Germany; 6 University Medical Center Mainz, Mainz, Germany; 7 Universitätsklinikum Schleswig-Holstein Campus Lübeck, Lübeck, Germany; 8 Medical Center Nürnberg Nord, Nürnberg, Germany; 9 Medical Clinic 3, Universitätsklinikum Bonn, Bonn, Germany; 10 DKD Helios Klinik Wiesbaden, Wiesbaden, Germany; 11 University Medical Center LMU München, Munich, Germany; 12 Klinikum Augsburg, Augsburg, Germany; 13 Robert Bosch Krankenhaus Stuttgart, Stuttgart, Germany; 14 University Medical Center Hannover, Hannover, Germany; 15 Present Address: Klinikum Kulmbach, Bavaria, Germany; 16 Present Address: Dpt. Hematology and Oncology, Comprehensive Cancer Center Innsbruck (CCCI), Innsbruck, Austria; 17 Present Address: Universätsklinikum Würzburg, Würzburg, Germany

**Keywords:** Phase IV trials, Leukaemia, Acute myeloid leukaemia

Correction to: *Bone Marrow Transplantation* 10.1038/s41409-024-02264-9, published online 16 March 2024

In this article the title was incorrectly given as ‘Long term results of a prospective multicenter obervational study on the use of anti-human T-lymphocyte immunoglobulin (ATLG) in unrelated donor transplantation (ATOS study)’ but should have been ‘Long term results of a prospective multicenter observational study on the use of anti-human T-lymphocyte immunoglobulin (ATLG) in unrelated donor transplantation (ATOS study)’.

Further in this article the affiliation details for Author Dominik Wolf were incorrectly given as ^16^Present address: Dpt. Hematology and Oncology, Comprehensive Cancer Center Innsburck (CCCI), Vienna, Austria but should have been ^16^Present address: Dpt. Hematology and Oncology, Comprehensive Cancer Center Innsburck (CCCI), Innsbruck, Austria.

The original article has been corrected.

