# Supplementary material for: Long term results of a prospective multicenter observational study on the use of anti-human T-lymphocyte immunoglobulin (ATLG) in unrelated donor transplantation (ATOS study)
Source: Bone Marrow Transplant. 2024 Mar 16;59(7):936–41. doi: 10.1038/s41409-024-02264-9 (PMC11226393; doi:10.1038/s41409-024-02264-9)
Supplement: Supplementary file 1 — ATOS supplement data [file 41409_2024_2264_MOESM1_ESM.docx]

**Supplementary appendix**

**Supplementary Methods**

**Study design and participants**

Patients had to fulfil the following inclusion criteria: Male or female patients, 18-65 years of age, written informed consent, suffering from hematological malignancies, and designated to undergo allogeneic bone marrow or peripheral blood stem cell transplantation from matched unrelated donors who will receive GvHD prevention according to the Summary of Product Characteristics (SPC) of ATLG. Patients who previously underwent allogeneic or autologous transplantation were not included. According to the SPC, for conditioning prior to stem cell transplantation as part of MAC regimens for allogeneic stem cell transplantation, the standard dose is 20 mg/kg/d, usually starting from day -3 to day -1 prior to SCT. The patients should only be considered for participating in this NIS after the decision was made to start therapy with ATLG. In order to avoid selection bias, physicians were asked to include all patients fulfilling the inclusion and exclusion criteria in a consecutive way. The NIS was conducted in 13 German transplant centers, 5 of them had also participated in our former RCT.

**Procedures**

No study specific visits were scheduled. Documentation was done at the routine visits and data were collected within the study at baseline at time of SCT, 100 days after SCT, and 12 months after SCT. Data collected were on engraftment, GvHD, relapse, survival, new malignancies, quality-of-life, employment status, hospital stays, and safety. Adverse drug reactions (ADRs) for which a causal relationship to the applied study drug was at least a reasonable possibility as well as severe infections independent of their causal relationship to the study drug were to be documented by the participating centers starting with the first administration of ATLG and until 12 months after SCT. All study relevant data were collected on paper case report forms which were created for this purpose. Clinical monitoring with on-site visits for source data verification was conducted for quality assurance. Patient data were pseudonymized and completed and signed CRFs were sent to the clinical trials unit for electronic data management. Besides the pseudonymization code the patients’ DRST (German Registry for Stem Cell Transplantation) identification codes were also collected to enable potential later comparisons and analyses with the DRST database (further details in Supplementary appendix).

**Statistical analysis**

The effects of the following prognostic factors on outcome were analyzed in univariate and multiple Cox regression models: patient age, donor age, patient and donor sex, patient and donor CMV status, type of primary disease, disease status, conditioning intensity, conditioning regimen, time from primary diagnosis of current disease to transplantation, stem cell source, HLA-mismatch, and extent of GvHD prophylaxis (ATLG dose). Special interest focussed on disease status, conditioning intensity, conditioning regimen, HLA-mismatch, and ATLG dose. In these analyses, other factors and center were included for adjustment if they were empirically related to the interesting factor. In general, prognostic factor analyses have to be interpreted with caution, as associations observed cannot be interpreted as causal effects. In univariate analyses, it may happen that an effect is seen or not seen just because correlations to other variables exist. Multiple regression analyses including many variables can have problems of instability in case of low event numbers. Furthermore, multiplicity issues exist, due to the large number of factors and endpoints. Keeping these limitations in mind, all results have to be interpreted in a descriptive and exploratory sense.

A sample size of 150 patients was deemed feasible for recruitment within the planned time period and necessary to obtain precise results. With 150 patients, the incidence of an event can be estimated with a minimal precision of ± 0.08 (maximal width of 95%-confidence interval if incidence is 0.5). The power for the evaluation of prognostic factors was calculated as follows. With respect to an endpoint with an incidence of 0.3 (0.5), leading to 45 (75) events, a binary prognostic factor with a prevalence of 0.5 can be detected with a power of 0.8 when its effect corresponds to a HR of 2.3 (1.9), respectively.

Additionally, the outcome of the patients of the ATOS study was compared to the outcome of the patients in the ATLG treatment arm in our RCT. In these analyses, comparisons were adjusted for patient age, donor age, disease status,^8^ conditioning intensity, stem-cell source, HLA-match, and extent of GvHD prophylaxis (ATLG dose).^1, 2^

**Supplementary Results**

**Clinical efficacy endpoints**

**Comparison to the ATLG arm in our RCT**^2^

The incidences and rates of events were compared to those observed in the ATLG arm of our RCT,^2^ where the median follow-up time had been 9.0 years (IQR 8.0-9.6 years). Incidences of acute GvHD, relapse, NRM, and rates of DFS, and OS were similar to the results obtained in our RCT Incidences of chronic GvHD (5 year: 0.42) and especially of severe chronic GvHD (5 year: 0.27) were higher as compared to the results obtained in the ATLG arm of our RCT (any 0.31, severe 0.14). As a consequence of these differences, the rate of SGRFS was somewhat lower in the ATOS study as compared to the ATLG arm in our RCT.

We tried to adjust for different patient and treatment characteristics in both studies in multiple Cox regression models including patient age, donor age, disease status, conditioning intensity, stem cell source, HLA-mismatch, and ATLG dose. The results are shown in table S2. All adjusted comparisons except for severe chronic GvHD resulted in 95%-CI of the hazard ratio overlapping the value of one. Figure S2 shows the SGRFS rates of ATOS vs the ATLG arm of the RCT estimated by Kaplan-Meier estimators (unadjusted) and from the Cox regression model (adjusted). The adjusted HR (ATOS vs ATLG RCT) was estimated as 1.25, 95%-CI (0.81-1.91), p=0.31. Figure S3 shows the OS rates of ATOS vs the ATLG arm of the RCT estimated by Kaplan-Meier estimators (unadjusted) and from the Cox regression model (adjusted). The adjusted HR (ATOS vs ATLG RCT) was estimated as 0.70, 95%-CI (0.41-1.18), p=0.18.

The interpretation of differences between both studies also has to take into account that differences in data collection procedures existed which are less relevant for objective endpoints as OS and which are more relevant for subjective endpoints as GvHD and relapse. The difference in the incidence of severe chronic GvHD could not be explained by differences in patient and disease characteristics with an adjusted hazard ratio (ATOS vs ATLG arm RCT) estimated as 2.79, 95%-CI (1.20-6.51), p=0.017. This may in part possibly be due to the fact, that chronic GvHD in the RCT was assessed solely according to the Seattle criteria, whereas chronic GvHD in ATOS and in the DRST were assessed according to the Seattle criteria and to the NIH criteria. But in general, in ATOS, with less standardized criteria for data evaluation, the assessments were heterogeneous, and we observed large differences in rates of relapse and of chronic GvHD between centers.

**Hospital stays, quality of life**

The median hospital stay for SCT was 38.0 days, readmission took in median 3.0 days, and the median number of visits in outpatient clinic within 12 months after SCT was 18 (available data for 150 patients). The median Karnofsky Index at month 12 after SCT of 115 patients with available data was 90.0%. Regarding QOL, data were available for 95 patients at month 12 after SCT. No problems with regard to mobility, self-care, usual activity, pain/discomfort, anxiety were reported for 82.1%, 91.6%, 63.2%, 48.9%, and 76.8%, respectively. After acute GvHD grade III-IV, patients reported more problems in some QOL aspects (mobility, self-care, usual activities) as compared to patients without severe acute GvHD, and patients with relapse up to month 12 reported more anxiety as compared to patients without relapse. After one year of follow-up the survival rate free of immunosuppressive therapy was 54.5 %, which may possibly be regarded as indication for normalization of quality of life after SCT. 23.3% of 129 patients for whom employment status was available were working.

**Adverse drug reactions, severe infections, reasons of death**

Interruption or discontinuation of ATLG administration occurred in nine patients due to adverse events; eight patients recovered, one patient deceased from sepsis which was judged by the investigator as not related to ATLG. There were no patients withdrawn from the study due to ADRs.

The most frequently reported ADRs were “infections and infestations” (68.5%) and “general disorders and administration site conditions” (44.2%). The most frequently reported severe infections in this study were “pneumonia” (13.3%) and “cytomegalovirus infection” (12.1%) (see table S3). Comparing the ATOS study with the ATLG arm of the RCT, no safety issues were detected which may contravene the overall safety profile of ATLG observed in the pivotal AP-AS-21-DE study.

47 (28.5%) of patients died within the period of analysis. The causes of death were the following: relapse (n=15; 9.1%), infections (n=21; 12.7%), aGvHD (n=3; 1.8%), secondary malignancy (n=2; 1.2%) and other causes (n=6; 3.6%).

**Supplementary Tables and Figures**

**Table S1**: Total given ATLG-dose by center

|  |  | Total given dose per kg [mg/kg] | | | |
| --- | --- | --- | --- | --- | --- |
| Center | N | Mean | Median | IQR | (Minimum, Maximum) |
| 01 | 40 | 32.2 | 32 | (31,34) | (15,36) |
| 02 | 5 | 41.8 | 44 | (30,45) | (29,61) |
| 03 | 1 | 35.2 | 35 | (35,35) | (35,35) |
| 04 | 4 | 60.0 | 60 | (60,60) | (60,60) |
| 05 | 24 | 51.4 | 59 | (37,60) | (28,62) |
| 06 | 13 | 46.0 | 59 | (30,60) | (24,62) |
| 08 | 11 | 30.8 | 30 | (30,32) | (28,36) |
| 09 | 5 | 55.2 | 60 | (50,60) | (46,60) |
| 10 | 14 | 60.3 | 60 | (60,61) | (57,62) |
| 12 | 8 | 50.7 | 58 | (40,60) | (30,61) |
| 15 | 4 | 59.8 | 60 | (59,61) | (58,61) |
| 20 | 6 | 54.8 | 60 | (59,60) | (30,60) |
| 21 | 30 | 53.7 | 59 | (40,60) | (25,91) |
| **Total** | **165** | **46.4** | **46** | **(32,60)** | **(15,91)** |

**Table S2**: Comparison of patients in the ATOS study with patients in the ATLG arm of our RCT with respect to different endpoints: Multiple Cox regression models adjusted for patient age, donor age, disease status, conditioning intensity, stem-cell source, HLA-mismatch, ATLG-dose

|  | ATOS vs. ATLG (RCT) | | | |
| --- | --- | --- | --- | --- |
| Endpoint | Hazard ratio | 95%-CI | | p-value |
| Severe GvHD and relapse-free survival (SGRFS) | 1.25 | 0.81 | 1.91 | 0.31 |
| Acute GvHD (any) | 0.92 | 0.58 | 1.44 | 0.71 |
| Acute GvHD (grade III-IV)) | 0.78 | 0.24 | 2.52 | 0.68 |
| Chronic GvHD (any) | 1.46 | 0.78 | 2.70 | 0.23 |
| Chronic GvHD (severe) | 2.79 | 1.20 | 6.51 | 0.017 |
| Relapse | 0.91 | 0.51 | 1.65 | 0.76 |
| Non-relapse mortality (NRM) | 0.53 | 0.23 | 1.25 | 0.15 |
| Disease-free survival (DFS) | 0.78 | 0.48 | 1.27 | 0.32 |
| Overall survival (OS) | 0.70 | 0.41 | 1.18 | 0.18 |

**Table S3**

Summary of all adverse drug reactions (ADR) and severe infections by system organ class and preferred term

|  | | **Incidence of ADR and severe infections** | |
| --- | --- | --- | --- |
| **System organ class** | **Preferred term** | **N** | **Total %** |
| Total number of patients |  | 165 | 100.0 |
| Number of patients with at least one ADR |  | 157 | 95.2 |
| Infections and infestations |  | 113 | 68.5 |
|  | Pneumonia | 22 | 13.3 |
|  | Cytomegalovirus infection | 20 | 12.1 |
|  | Sepsis | 16 | 9.7 |
|  | Device related infection | 13 | 7.9 |
|  | Oral herpes | 13 | 7.9 |
|  | Infection | 9 | 5.5 |
| General disorders and administration site conditions |  | 73 | 44.2 |
|  | Pyrexia | 67 | 40.6 |
|  | Chills | 27 | 16.4 |
| Investigations |  | 62 | 37.6 |
|  | Blood bilirubin increased | 51 | 30.9 |
|  | Blood creatinine increased | 10 | 6.1 |
| Blood and lymphatic system disorders |  | 52 | 31.5 |
|  | Febrile neutropenia | 44 | 26.7 |
| Gastrointestinal disorders |  | 40 | 24.2 |
|  | Nausea | 11 | 6.7 |
|  | Stomatitis | 11 | 6.7 |
|  | Diarrhea | 10 | 6.1 |
| Vascular disorders |  | 19 | 11.5 |
|  | Hypotension | 10 | 6.1 |
| Cardiac disorders |  | 17 | 10.3 |
| Hepatobiliary disorders |  | 15 | 9.1 |
| Skin and subcutaneous tissue disorders |  | 15 | 9.1 |
| Respiratory, thoracic and mediastinal disorders |  | 11 | 6.7 |
| Nervous system disorders |  | 10 | 6.1 |
| Renal and urinary disorders |  | 10 | 6.1 |

**Figure S1:** Estimated probability of overall survival (OS) by center and ATLG-dose - unadjusted and from Cox regression model adjusted for patient age, donor age, CMV status of patient, type of disease, disease status, time from primary diagnosis of current disease to transplantation, HLA-mismatch

**Figure S2** Estimated probability of severe GvHD and relapse-free survival (SGRFS) by study

**Figure S3**: Estimated probability of overall survival (OS) by study
